# A meta-analysis comparing 48-week treatment outcomes of single and multi-tablet antiretroviral regimens for the treatment of people living with HIV

**DOI:** 10.1186/s12981-018-0204-0

**Published:** 2018-10-30

**Authors:** Patrick G. Clay, Wei C. Yuet, Christiane H. Moecklinghoff, Inge Duchesne, Krzysztof L. Tronczyński, Sandip Shah, Dong Shao

**Affiliations:** 1 University of North Texas System College of Pharmacy, 3500 Camp Bowie Blvd, Fort Worth, TX 76107 USA; 2 Jannsen-Cilag GmbH, Johnson & Johnson Platz 1, 41470 Neuss, Germany; 3 Janssen EMEA, Turnhoutseweg 30, 2340 Beerse, Belgium; 4 Janssen-Cilag Polska Sp. Z o.o, Iłżecka 24, 02-135 Warsaw, Poland; 5 Market Access Solutions, LLC, 575 NJ-28, Raritan, NJ 08869 USA

**Keywords:** Human immunodeficiency virus, Treatment adherence and compliance, Treatment outcome, Quality of life, Economics pharmaceutical

## Abstract

**Objectives:**

To compare outcomes with single tablet regimens (STR) versus multi-tablet regimens (MTR) for human immunodeficiency virus (HIV) treatment using published data.

**Design:**

Systematic review and random-effects meta-analysis of literature on approved and investigational HIV regimens.

**Methods:**

The research followed the Preferred Reporting Items for Systematic Reviews and Meta-Analyses guidelines. Single or un-blinded studies reporting a direct comparison between STR and MTR were eligible for the meta-analysis. Double-blinded studies were excluded due to lack of difference in pill burden between cohorts. The key outcomes of interest included: adherence rates/proportion meeting target, efficacy, safety/tolerability, non-clinical and economic outcomes.

**Results:**

After screening 63 full-text articles and posters, 14 studies were eligible for the meta-analysis. The analysis showed that patients taking STR had improved outcomes over those taking MTR. Patients were significantly more adherent regardless of daily dosing frequency (odds ratio [OR]: 1.96, p < 0.001) and were more likely to achieve virological suppression (relative risk [RR]: 1.05, p = 0.002). There was a trend toward a lower discontinuation risk in the STR cohort, together with reported higher therapy satisfaction, better symptom control, improved health status, reduced healthcare resource utilization and demonstrated cost-effectiveness compared to MTR. There were no differences in CD4 cell count increase (at 48 weeks) or safety outcomes.

**Conclusions:**

The findings of this study confirm previously reported preliminary findings of the advantages of STR over MTR for HIV treatment in adherence, therapy continuation, viral suppression, tolerability, quality of life improvement, cost-effectiveness and healthcare resource utilization.

**Electronic supplementary material:**

The online version of this article (10.1186/s12981-018-0204-0) contains supplementary material, which is available to authorized users.

## Introduction

Although treatment options have expanded significantly, human immunodeficiency virus (HIV) remains an important global public health issue. In 2017, the number of people living with HIV (PLWH) was approximately 36.9 million [1] and about 1.1 million people died of acquired immune deficiency syndrome (AIDS)-related illnesses [1]. The annual rate of new infections remained relatively constant between 2005 and 2017, however, the number of PLWH receiving antiretroviral therapy (ART) increased dramatically from 2.2 million to 21.7 million [1].

ART has evolved significantly in the past two decades. Beginning with protease inhibitors in 1995, through multiple drug single class combination tablets in 2001 and multiple drug, multiple class combination tablets in 2006, to the most recent and sixth ART class, post-attachment inhibitors (ibalizumab), in 2018. Today, multiple class, multiple drug, fixed-dose, single tablet regimens (STR) dominate use [2]. Collectively, these advances have improved outcomes, have enhanced tolerability and reduced pill burden and enabled more PLWH to reach goal adherence levels [3]. With each newly approved STR, there is potential for treatment guidelines/practice patterns to change in PLWH naïve to therapy, yet the needs of the individual is paramount [4, 5]. Efforts continue to develop long-acting mechanisms to deliver ART that may improve durability [3, 6].

Simplified regimens improve adherence and clinical outcomes and have long been proven in other conditions, such as hypertension, diabetes and asthma [3, 7]. With the advent of STR, HIV has become a treatable, chronic disease [8] with both the Joint United Nations Programme on HIV and AIDS (UNAIDS) and the World Health Organization (WHO) highly recommending once daily fixed-dose combinations (FDC) to improve adherence [6, 9].

FDC can include multiple classes of ART and may represent an entire recommended regimen taken once daily (e.g., one ‘tablet’ once daily). FDC reduce the number of total daily pills (pill burden) and contain at least two active agents, rather than a single dosing unit with a single active agent with a pharmacokinetic enhancer/booster, such as Kaletra™ (Iopinavir/ritonavir), Prezcobix™ (darunavir/cobicistat [DRV/COBI]) and Evotaz™ (atazanavir/cobicistat [ATV/COBI]) [10].

When a FDC constitutes an entire regimen and is provided as one ‘tablet’ for use once daily, it is known as an STR. However, FDC can be co-administered with another ART agent to create a multiple-tablet regimen (MTR). MTR can be taken once or more times per day, dependent upon the MTR components. Since 2014, a number of STR and MTR have been approved and are now recommended by treatment guidelines as first-line treatment for treatment-naïve patients. The newly included STR regimens include elvitegravir/cobicistat/emtricitabine/tenofovir alafenamide (EVG/COBI/FTC/TAF), darunavir/cobicistat/emtricitabine/tenofovir alafenamide (DRV/COBI/FTC/TAF) and emtricitabine/rilpivirine/tenofovir alafenamide (FTC/RPV/TAF). MTR component agents include ATV/COBI, DRV/COBI and emtricitabine/tenofovir alafenamide (FTC/TAF). As none of these combinations have been compared in a systematic review [11], the objective of this study is to compare the clinical (adherence, virologic and safety/tolerability), economic and non-clinical or humanistic outcomes of all currently available STR and MTR.

## Methods

### Search strategy and study selection

A literature review and meta-analysis was conducted to include all currently approved FDC, both STR and MTR. The study followed the Preferred Reporting Items for Systematic Reviews and Meta-Analyses (PRISMA) guidelines and the PICOS principles (Patient, Intervention, Comparator, Outcome, and Study Design) based on an internal study protocol (available upon request) [12]. Databases including Embase, PubMed and ClinicalTrials.gov were searched to capture relevant literature published from 2005 to 2017, with filters to include only studies conducted in humans and published in English.

The endpoints of interest included adherence, efficacy, safety and healthcare resource utilization along with an emerging element for decision makers, humanistic outcomes [11]. Data included published, publicly available randomized controlled trials (RCT) and observational studies. Case studies, abstracts without posters or full-text, letters, reviews, editorials and comments were excluded. Two reviewers conducted a two level screening process. A first-pass screening of bibliographic details, titles and abstracts of all citations eliminated duplicates and irrelevant studies. Full-text of studies meeting the eligibility criteria and reported outcomes of interest were included for data extraction and screened for inclusion. For all quantitative outcomes, only studies with outcome measures in evaluable format (n/N, mean, standard deviation, N or median and inter-quartile range) with a clear comparison between STR and MTR arms were included. Double-blind studies were excluded as enrolled subjects would receive the same number of tablets, thus removing direct comparison between MTR and STR. Qualitative evidence data capture reporting in the literature precluded use of this standard, thus data was included when provided and resulted in slight variance in studies used. For all studies however, the methodological quality of RCTs was assessed using the Cochrane handbook, focusing on the risk of bias across five different categories (selection, performance, detection, reporting and attrition) [13]. For observational studies, the Critical Appraisal Skills Programme (CASP) Cohort Study Checklist was used to evaluate the overall study quality [14]. A random-effects meta-analysis was conducted using Comprehensive Meta-Analysis Software (version 2) for data analysis and to create the forest plots.

### Endpoints

The 48-week primary endpoints were used in all analyses. Adherence outcomes included achieving a protocol specific threshold measure (yes/no, dichotomous) and as author reported adherence frequency (pill count, percentage adherence or proportion of days covered, etc.). Efficacy outcomes included the percentage of patients achieving viral load suppression (i.e., < 50 copies/ml) and changes in mean CD4 counts from baseline. Safety outcomes included the percentage of patients experiencing any severe adverse event (SAE), mortality or any grade 3 to 4 clinically significant event or laboratory abnormality. Tolerability outcomes included the percentage of patients discontinuing their STR or MTR for any reason. Economic and humanistic outcomes are summarized in the review to provide a single-source review of STR compared with MTR to inform healthcare providers as well as policymakers.

### Statistical methods

Inverse variance methods were used in a random-effects model to analyze both dichotomous and continuous data and to assess heterogeneity [15]. Heterogeneity was evaluated using the Chi squared test and quantified using the I^2^ statistic [16]. Alpha < 0.05 was used to determine statistical significance. I^2^ values of 25, 50 and 75% correspond to low, medium and high levels of heterogeneity, respectively. Summary statistics were calculated for each study to describe observed treatment effects; mean and standard deviation values were calculated where studies reported median and inter-quartile range. When necessary, 95% confidence intervals (CI) were converted to standard deviations via the formula SD = (√N)*((upper limit − lower limit)/3.96). A pooled treatment effect estimate was then calculated as the weighted average of the treatment effects estimated in the individual studies. Each study was weighted as the inverse of the variance of the effect estimate (i.e., one over the square of its standard error). Larger studies with smaller standard errors were given more weight than smaller studies with larger standard errors. For the studies which had multiple MTR arms, data from the MTR arms were first pooled within the trials and then between the trials. Dichotomous outcomes were evaluated by making an adjustment to the study weights according to the extent of variation, or heterogeneity, among the treatment effects.

Values for dichotomous outcomes (adherence [based on a threshold measure; yes/no], viral load suppression, safety events, and tolerability) were presented as n/N, where n = subset of sample size; N = total sample size, and the odds ratio (OR) or risk ratio (RR) with 95% CI were calculated. Values for continuous outcomes (CD4 cell counts and adherence [based on pill count or percentage of drug(s) used]) were presented as mean, standard deviation (SD) and N (sample size), with calculated standardized mean differences. For humanistic (qualitative) data, a global approach was adopted to rate the results as negative, neutral or positive based on the change in scores from baseline to the end of the study period. For economic evaluations where studies reported healthcare resource use (HRU), the direct medical costs and incremental cost-effectiveness ratio (ICER) values were summarized.

## Results

### Study details

**Fig. 1:**
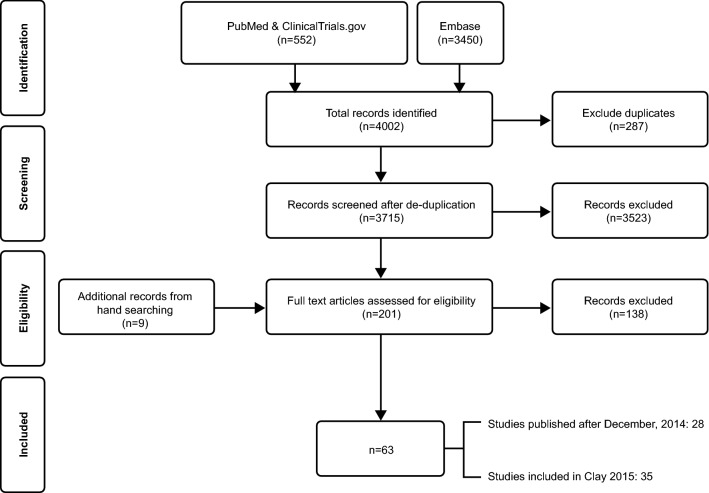
PRISMA flow diagram for literature search and study selection

The literature search yielded 4002 citations, of which 287 were duplicates, resulting in 3715 unique records. After screening titles and abstracts, 192 potentially relevant studies were identified. Nine additional articles were found from hand searching of bibliographies. Following a careful examination of the 201 full-text articles and clinicaltrials.gov records, a total of 63 studies (34 RCT, 24 observational studies (OS) and five economic studies) met the inclusion and exclusion criteria and were included for qualitative evidence synthesis. The PRISMA flow of the review process is shown in Fig. 1. Nearly half (n = 30) of the studies were conducted in treatment naïve PLWH, 22 studies included treatment experienced PLWH and 11 included both treatment experienced and naïve PLWH.

Twenty-eight of the 63 studies were not included in the previous meta-analysis [11]. Most of the studies not included in the previous review reported efficacy (73%) and safety/tolerability (59%). Adherence was reported in 48% of the added studies. Fewer reported (or assessed) humanistic measures (patient-reported outcomes [PRO]) (14%) and economic endpoints (21%).

Key characteristics of the 63 studies are summarized in Additional file 1: Table S1 Of these 63 studies, only 14 studies reported outcome measures in evaluable format (n/N, mean, standard deviation, N, or median and inter-quartile range) and/or at consistent evaluation time points, with a clear comparison between STR and MTR, and were included for meta-analysis. Baseline demographics of the population in the 14 studies are shown in Additional file 2: Table S2.

The majority of the included RCTs had low risk of bias, with potentially high risk for blinding in treatment allocations. The overall quality of all observational studies was determined as medium and satisfactory. The detailed assessments are presented in Additional file 3: Table S3.

### Adherence outcomes

While 30 of the 63 studies reported patient adherence outcomes, only eight studies reported quantifiable data and were included in the meta-analysis [17–24]. Seven of these eight studies [17–23] reported patient adherence as a dichotomous outcome (threshold defined per study protocol), two reported mean difference in medication adherence calculated using pill count and one reported using both formats [17, 24].

**Fig. 2:**
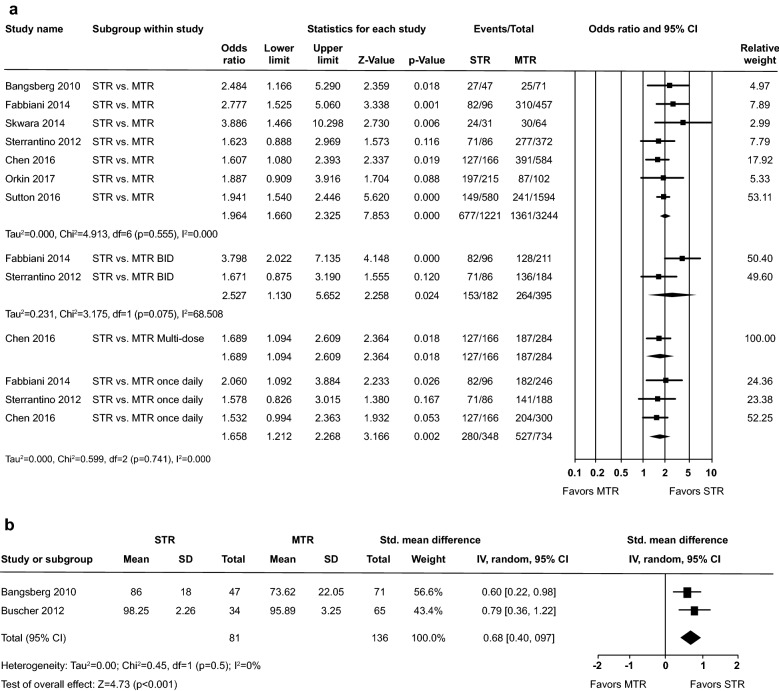
**a** Adherence rate (dichotomous measure) and **b** adherence per pill count

In the dichotomous adherence outcome analysis, adherence was significantly better in patients receiving STR than patients receiving once or twice daily MTR: 55.4% (range: 25.7% to 91.6%) versus 42.0% (range: 15.1% to 85.3%), odds ratio (OR) of adherence: 1.96, 95% confidence interval (CI) 1.66–2.33, p < 0.001. No heterogeneity was observed (Chi^2^ = 4.91; i^2^ = 0.0%) (Fig. 2a).

In the sub-analysis of STR versus once daily MTR, adherence was significantly better in patients receiving STR compared with those receiving once daily MTR: 80.5% (range: 76.5% to 85.4%) versus 71.8% (range: 68.0% to 75.0%), OR: 1.66, 95% CI 1.21–2.27, p = 0.002; Fig. 2a).

In the sub-analysis of STR versus twice daily MTR, adherence was numerically better in patients receiving STR compared with those receiving twice daily MTR: 84.1% (range: 82.6% to 85.4%) versus 66.8% of patients (range: 60.7% to 73.9%), OR: 2.53, 95% CI 1.13–5.65, p = 0.02; Fig. 2a).

Medication adherence based on ‘‘pill count’’ (two studies) was higher in the STR group (92.1% [range: 86.0% to 98.3%]) compared with 84.8% (range: 73.6% to 95.9%) in the collective MTR groups. The standardized mean difference (SMD) comparing medication adherence was also statistically significantly in favor of the STR group (SMD: 0.68, 95% CI 0.40–0.97, p < 0.001) in the two studies (Fig. 2b).

### Efficacy outcomes

**Fig. 3:**
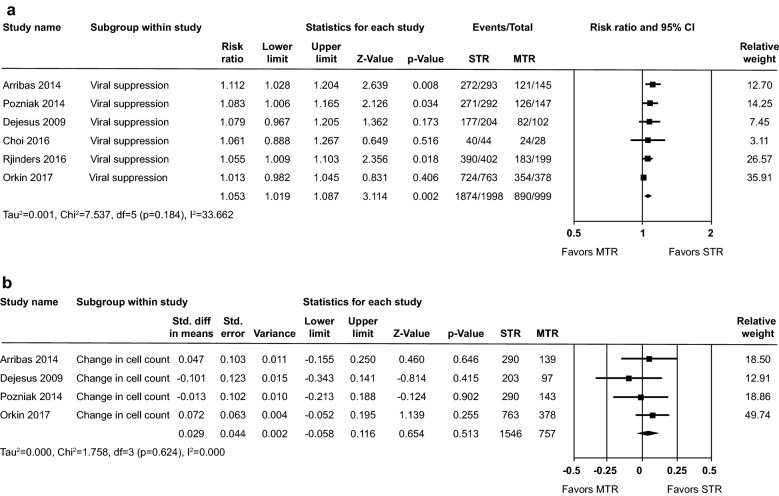
**a** Viral suppression (< 50 copies/ml) at 48 weeks and **b** Increase in CD4 cell count from baseline to 48 weeks

Twenty-four of the 63 studies reported efficacy data for viral load suppression and CD4 count. Eighteen studies were excluded from the meta-analysis since they reported time points other than 48 weeks or parameters not in a quantifiable format. Six studies [22, 25–29] provided analyzable data for viral load suppression (defined per protocol < 50 copies/ml) at 48 weeks and four studies [25–27, 30, 31] reported change in CD4 cell count at 48 weeks. There was a statistically significant difference in viral load suppression at 48 weeks between the STR and MTR groups (relative risk (RR): 1.05, 95% CI 1.02–1.09, p = 0.002) and low heterogeneity between the studies was observed (Chi^2^ = 7.54; i^2^ = 33.7%) (Fig. 3a). The difference (SMD) in CD4 cell count between STR and MTR was not statistically significant at 48 weeks (SMD: 0.029, 95% CI: − 0.06–0.12, p = 0.51), and no heterogeneity between the studies was observed (Chi^2^ = 1.76; i^2^ = 0.0%, Fig. 3b).

### Safety and tolerability outcomes

Of the 63 studies, 37 reported safety outcomes with data relevant to adverse events (AE), laboratory abnormalities, mortality, and tolerability (treatment discontinuation). Six studies [22, 25–28, 32] reported analyzable data for the safety and tolerability outcome parameters. All six studies [22, 25–28, 32] reported rates of discontinuation due to any reason, three reported protocol-defined SAE [22, 25, 26], five reported Grade 3 to 4 AE [22, 25, 26, 28, 32], two reported Grade 3 to 4 laboratory abnormalities [25, 32] and two reported mortality [25, 26].

**Fig. 4:**
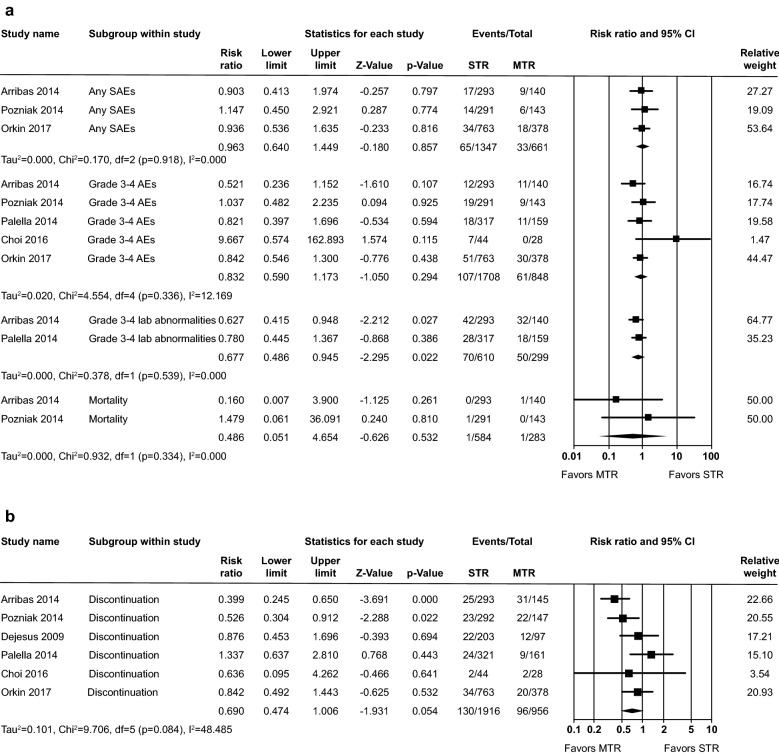
**a** Safety outcomes and **b** Discontinuation due to any reason

Meta-analyses of SAE, grade 3 to 4 AE and mortality revealed no statistically significant differences between STR and MTR groups (Fig. 4a) with RR of any SAE (RR: 0.96, 95% CI 0.64–1.45, p = 0.86), Grade 3 to 4 AE (RR: 0.83, 95% CI 0.59–1.17, p = 0.29) and mortality (RR: 0.49, 95% CI 0.05–4.65, p = 0.53) and minimal heterogeneity among the studies (Chi^2^ = 0.17–4.55; i^2^ = 0.00–12.17%). Grade 3 to 4 laboratory abnormalities were significantly less likely for the STR group versus the collective MTR groups, RR: 0.68, 95% CI 0.49–0.94, p = 0.02, with no heterogeneity in the studies.

The risk of discontinuation due to any reason was lower in the STR group versus the MTR group, RR: 0.69, 95% CI 0.47–1.00, p = 0.05, —. 4B. Moderate heterogeneity was observed in the tolerability studies (Chi^2^ = 9.71, i^2^ = 48.49%), potentially due to variation in study design and/or population.

### Humanistic or patient-reported outcomes (PRO)

Seven [19, 20, 25, 27, 33–35] of the 63 studies reported evaluable results of PRO associated with ART. The most commonly used instruments in the studies included the HIV Treatment Satisfaction Score (HIV-TSQ), HIV Symptom Index (HIV-SI) and the 36-Item Short Form Health Survey (SF-36). Variability among the instruments used meant that the meta-analysis could not include PRO data. Five studies [25, 27, 33–35] reported positive impact with results favoring STR in patient satisfaction, symptom control and overall health status. Among these five studies, the impact of STR or MTR on mental health was rated as neutral. Among the five, only one directly compared STR and MTR [25]. In this study, STR was associated with a higher HIV-TSQ score as compared to MTR at weeks 4 (21.5 versus 13.3, p < 0.0001) through week 24 (23.1 versus 14.5, p < 0.0001) [25]. Further, STR resulted in lower rates of patient-reported AE such as diarrhea (30% versus 46%, p < 0.001) and bloating (33% versus 41%, p = 0.039) compared with MTR (p < 0.05 for both) [25]. Similar improvements in the STR and MTR arms in the other two studies, regardless of pill burden [19, 20].

### Economic summary

Thirteen [23, 36–47] of 63 studies reported economic outcomes. Ten studies were summarized in the previous review [11]. Three additional studies were identified and are reported in this publication [23, 44, 45]. One study, evaluating overall ART changes in 3850 PLWH, found that modifying therapy resulted in a mean additional cost of €14 (SD €216; range −€528 to +€831) per month per patient [44]. Toxicity and therapy simplifications were cited as the leading causes for regimen changes [44]. Economic outcomes have been modeled using simulations and insurance claims data including comprehensive computer-based microsimulation to compare the cost-effectiveness of STR to MTR for initial treatment and concluded that the ICER of STR to MTR is $26,383 per quality-adjusted life year [45]. A multivariate regression model study using Medicaid medical and pharmacy claims data was completed to determine the impact of ART pill burden in 2174 PLWH [23]. Patients taking STR had a lower risk of hospitalization (HR: 0.71, 95% CI 0.59–0.86, p = NS) and extended time to hospitalization (median: 1508 versus 1032 days, p = 0.0042) [23].

## Discussion

The results with respect to adherence, viral load, Grade 3 to 4 laboratory abnormalities favor STR versus MTR. However, in contrast to previous review, the risk of discontinuation due to any reason was lower in the STR group, implying a better tolerability profile. Improved adherence and low discontinuation rates have been seen in other conditions when treatments are simplified that could be attributed to lower pill burdens and dosing frequencies [7]. Although the study was not designed to address the issue, it may be that patient satisfaction with STR may contribute to better adherence and lower rates of discontinuation, leading to improved clinical outcomes. This aspect, rarely reported in clinical trials, should be given enhanced consideration for inclusion.

Improved clinical outcomes could in turn result in lower resource utilization and cost effectiveness of therapy. In fact, the economic studies included in this update indicated lower risk of hospitalization, as well as an ICER of approximately $26,000, which is within the range of willingness to pay thresholds [45]. It should be noted that these economic findings are based on OS and modelling, with inherent selection bias based on variations in patient characteristics and multiple assumptions which may or may not accurately reflect the treatment paradigm in a real-world setting.

We also examined humanistic outcomes based on patient reported data (not included in the meta-analysis), which was not carried out in the previous review. The findings were by and large positive for STR with respect to better patient satisfaction, symptom control and health status, including a lower rate of diarrhea and bloating. Given the weight that humanistic outcomes are gaining with third party payers and government funding bodies, it is worth considering when deciding regimen options. Further, consistent use of assessment tool or inclusion of multiple tools to permit higher order analyses is encouraged.

Similar to the previous review, the strengths of this update are based on the implementation of a well-defined search strategy and the use of a robust random-effects model to assess pooled estimates extracted from both RCTs and observational studies. We only included studies which had quantifiable outcomes that could be included in an analysis. For this reason, we only included open label studies with 48-week outcomes data for the meta-analysis, to enable us to compare STR and MTR. We excluded double blinded, randomized studies where patients on STR would also receive placebo pills (to equal the total number of pills for patients on MTR), since this would not result in an accurate comparison. Authors acknowledge that different classes of medications were compared and factors such as the mechanism of action might also play a role in safety and efficacy results.

## Conclusion

Patients on STR have better adherence, lower rates of discontinuation, improved viral load and fewer laboratory abnormalities than those on MTR. Economic and humanistic outcomes favor STR. Additional studies to examine the link between patient satisfaction, adherence and clinical outcomes in PLWH receiving STR or MTR, would provide additional evidence to support STR over MTR. In the meantime, it is important to implement and follow current global and country specific guidelines that specify STR as one of the primary recommended treatments in the management of PLWH. Improving access to STRs for patients, physicians and healthcare systems is critical, in improving the quality of life of PLWH.

## Additional files

**Additional file 1.** Characteristics of studies included in qualitative evidence synthesis.

**Additional file 2.** Characteristics of study population included in the meta-analysis.

**Additional file 3.** a. Quality of RCTs Included in quantitative evidence synthesis (studies included in meta-analysis). b. Quality of observational studies included in quantitative evidence synthesis (studies included in meta-analysis).
