# Supplementary material for: A meta-analysis comparing 48-week treatment outcomes of single and multi-tablet antiretroviral regimens for the treatment of people living with HIV
Source: AIDS Res Ther. 2018 Oct 30;15:17. doi: 10.1186/s12981-018-0204-0 (PMC6206661; doi:10.1186/s12981-018-0204-0)
Supplement: Supplementary file 1 — Additional file 1. Characteristics of studies included in qualitative evidence synthesis. [file 12981_2018_204_MOESM1_ESM.docx]

**Table S1: Characteristics of studies included in qualitative evidence synthesis.**

| **Study** | **Study Type** | **Intervention** | **Comparator** | **Population** | **Sample Size** | **Outcomes assessed** | **Included in meta-analysis?** | |
| --- | --- | --- | --- | --- | --- | --- | --- | --- |
| Arribas et al [1] | RCT | EVG/COBI/TDF/FTC | TDF/FTC þ RTV-boosted PI | TE | 438 | Adherence, efficacy, safety/tolerability | Yes (efficacy, safety/tolerability) | |
| Cohen et al [2] | RCT | EVG/COBI/TDF/FTC | EFV/TDF/FTC | TN | 71 | Efficacy, safety/tolerability | No | |
| Cohen et al [3] | RCT | RVP/TDF/FTC | RVP/TDF/FTC | TN | 799 | Adherence, efficacy, safety/tolerability | No | |
| Dejesus et al [4] | RCT | EFV/TDF/FTC | PI (with or without RTV boosting) + at least 2  NRTIs; or NNRTI + at least 2 NRTIs | TE | 306 | Adherence, efficacy, safety/tolerability | Yes (efficacy, safety/tolerability) | |
| Landman et al [5] | RCT | EFV/TDF/FTC | TDF/FTC + NVP; TDF + LPV/r; TDF/FTC + AZTC | TN | 120 | Adherence, efficacy, safety/tolerability | No | |
| NCT00112047 [6] | RCT | EFV/TDF/FTC | CBV þ EFV | TN | 511 | Efficacy, safety/tolerability | No | |
| NCT01403051 [7] | RCT | EFV/TDF/FTC | EFV/FTC/TDF plus vitamin D3 and calcium carbonate | TN | 167 | Efficacy, safety/tolerability | No | |
| Palella et al [8] | RCT | RVP/TDF/FTC | RTV-boosted PI + 2 NRTIs | TE | 482 | Adherence, efficacy, safety/tolerability | Yes (safety/tolerability) | |
| Pozniak et al [9] | RCT | EVG/COBI/TDF/FTC | NNRTI (EFV and non-EFV) + TDF/FTC | TE | 439 | Adherence, efficacy, safety/tolerability | Yes (efficacy,  safety/tolerability) | |
| Rockstroh et al [10] | RCT | EVG/COBI/TDF/FTC | RTV/ATV + TDF/FTC | TN | 715 | Adherence, efficacy, safety/tolerability | No | |
| Sax et al [11] | RCT | EVG/COBI/TDF/FTC | EFV/TDF/FTC | TN | 707 | Adherence, efficacy, safety/tolerability | No | |
| Sax et al [12] | RCT | EVG/COBI/FTC/TAF | EVG/COBI/FTC/TDF | TN | 171 | Adherence, efficacy, safety/tolerability | No | |
| Walmsley et al [13] | RCT | DRV + ABC/3TC | EFV/TDF/FTC | TN | 844 | Adherence, efficacy, safety/tolerability | No | |
| Airoldi et al [14] | RCT | EFV/TDF/FTC | Switched from 3TC/TDF or TFC/TDF FDCs (or combination of separate pills) | TE | 212 | Adherence, efficacy | No | |
| Bangsberg et al [15] | OS | EFV/TDF/FTC | RTV-boosted PI or NNRTI + 2 NRTIs | TN and TE | 118 | Adherence, efficacy | Yes (adherence) | |
| Beck et al [16] | OS | EFV/TDF/FTC | TDF/FTC + EFV; TDF + FTC + EFV; TDF + 3TC + EFV | TN | 1448 | HRU/costs | No | |
| Buscher et al [17] | OS | EFV/TDF/FTC | Any cART (incl. FDCs), with >1 pills | TN | 184 | Adherence, efficacy | Yes (adherence) | |
| Cohen et al [18] | OS | EFV/TDF/FTC | Any cART (incl. FDCs), with >1 pills | TN and TE | 7381 | Adherence, HRU/costs | No | |
| Colombo et al [19] | OS | EFV/TDF/FTC | EFV þ TDC þ FTC, ATV/r þ TDF þ FTC,  DRV/r þ TDF þ FTC, LPV/r þ TDF þ FTC | TN | 474 | Costs | No | |
| Engsig et al [20] | OS | TDF + 3TC + EFV | EFV/TDF/FTC | Group 1: TN Group 2: TE | Group 1: 167 Group 2: 868 | Efficacy | No | |
| Fabbiani et al [21] | OS | EFV/TDF/FTC | EFV + NRTI backbone (incl. TDF/FTC,AZT/3TC) | TN and TE | 553 | Adherence, efficacy | Yes (adherence) | |
| Grimes et al[22] | OS | EFV/TDF/FTC | ATZ/r + TDF/FTC, DRV/r + TDF/FTC, RAL + TDF/FTC, EFV + ABC/3TC, DRV + ABC/3TC, etc. | TN and TE | NA | Costs | No | |
| Hanna et al[23] | OS | EFV/TDF/FTC,  EVG/COBI/TDF/FTC,  RPV/TDF/FTC | Multiple-tablet regimen of any type | TE | 1727 | Adherence, efficacy | No | |
| Hill et al [24] | OS | EFV/TDF/FTC | LPV/r + TDF/FTC, LPV/r + AZT/3TC, Nevirapine + AZT/3TC, Nelfinavir + AZT/3TC, etc. | TN and TE | 115 | Efficacy | No | |
| Homar et al[25] | OS | EFV/TDF/FTC,  AZT/3TC, ABC/3TC,  ABC/AZT/3TC, TDF/FTC | PI or NNRTI-based cART with individual components | TE | 225 | HRU/costs | No | |
| Juday et al [26] | OS | EFV/TDF/FTC | Pi- or NNRTI-based cART with at least 2 NRTIs | TE | 461 | Adherence, HRU | No | |
| Juday et al [27] | OS | EFV/FTC/TDF,  EFV-containing regimen,  other NNRTI-containing regimen | LPV/r-containing, ATV/r-containing, other PI-containing regimen with TDF/FTC, AZT/3TC, etc. | TN | 2460 | Persistence | No | |
| Pujari et al [28] | OS | EFV/TDF/FTC | NA | TN | 141 | Adherence, efficacy, safety/tolerability | No | |
| Scourfield et al [29] | OS | EFV/TDF/FTC | NA | TN | 472 | Efficacy, safety/tolerability | No | |
| Skwara et al [30] | OS | EFV/TDF/FTC, RVP/TDF/FTC | Any cART (incl. FDCs), with >1 pills | TE | 95 | Adherence, efficacy, safety/tolerability | Yes (adherence) | |
| Sterrantino et al [31] | OS | PI-based and NNRTI-based  regimen, with NRTI  backbone (incl. FDCs) | EFV/TDF/FTC | TN and TE | 427 | Adherence, efficacy | Yes (adherence) | |
| Angeletti et al [32] | EM | NNRTI-based STR | RTV-boosted PI-based monotherapy or triple  regimen | TN and TE | NA | Costs | No | |
| Colombo et al [33] | EM | RPV/TDF/FTC | TDF/FTC + RPV, TDF/FTC + EFV, ABC/3TC + EFV, TDF/FTC + ATV/r, ABC/3TC  + ATV/r, TDF/FTC + DRV/r, TDF/FTC + RAL | TN | NA | Cost, QALYs, ICER | No | |
| Colombo et al [34] | EM | EFV/TDF/FTC | TDF/FTC + EFV | TN | NA | Costs, QALYs, ICER | No | |
| Walensky et al [35] | EM | No ART; EFV + 3TC + TDF | EFV/TDF/FTC | TN and TE | NA | Cost, QALYs, ICER | No | |
| **Studies published since 2015** | | | | | | | |  |
| Arribas et al [36] | RCT | E/C/F/TAF | E/C/F/TDF | TN | 1733 | Efficacy, safety/tolerability | No | |
| Sorbrino-Jimenez et al [37] | OS | NA | NA | TE | 92 | Reasons for switching and associated costs | No | |
| Choi et al [38] | RCT | E/C/F/TDF | EFV/F/TDF, ATV+RTV+F/TDF,  PI+RTV+ TVD, NNRTI+TVD | TN and TE | 72 | Efficacy, safety/tolerability | Yes (efficacy, safety/tolerability) | |
| Dejesus et al [39] | RCT | RPV/FTC/TAF | EFV/FTC/TDF | TE | 875 | Efficacy, safety/tolerability | No | |
| Gallant et al [40] | RCT | ATV, c, FTC/TDF | RTV, ATV, FTC/TDF. | TN | 692 | Efficacy, safety/tolerability | No | |
| Jiménez-Galán et al [41] | OS | EFV/FTC/ TDF or RPV/FTC/TDF | PI/r, PI/r plus one other drug | TE | 244 | Adherence | No | |
| Lewis et al [42] | OS | TDF/FTC, ABC/3TC+DRV/r, ATV/r, EFV or RAL, EFV/TDF/FTC, (RPV)/TDF/FTC | | TN | 1949 | Adherence | No | |
| Mills et al [43] | RCT | DRV/c/FTC/TAF + matched placebo | DRV/c + FTC/TDF + matched placebo | TN | 153 | Adherence, safety/tolerability | No | |
| Rijnders et al [44] | RCT | EVG/c/TAF/FTC | ATV boosted + FTC/TDF | TE | 601 | Efficacy,  safety/tolerability | Yes (efficacy) | |
| Mills et al [45] | RCT | EVG + c + TAF + FTC | TDF containing MTR | TE | 1443 | Efficacy,  safety/tolerability | No | |
| Orkin et al [46] | RCT | RPV/FTC/TAF | RPV/FTC/TDF | TE | 630 | Efficacy,  safety/tolerability | No | |
| Orkin et al [47] | RCT | EVG/c/TAF/FTC | EVG/c/TDF/FTC | TN | 1733 | Efficacy,  safety/tolerability | No | |
| Post et al [48] | RCT | FTC/TAF + boosted PI | FTC/TDF + unboosted third agent | TE | 663 | Efficacy,  safety/tolerability | No | |
| Rijnders et al [49] | RCT | EVG/c/TAF/FTC | EVG/c/TDF/FTC | TN | 1733 | Safety/tolerability | No | |
| Sax et al [50] | RCT | EVG/c/TAF/FTC | EVG/c/TDF/FTC | TN | 1733 | Efficacy,  safety/tolerability | No | |
| Squires et al [51] | RCT | EVG + c + TDF + FTC | RTV + ATV + FTC + TDF | TN | 575 | Adherence, efficacy, safety/tolerability,  PRO (no results) | No | |
| Sutton et al [52] | OS | EFV/FTC/TDF, FTC/RPV/TDF, or EVG/c/TDF/FTC | Various MTR | TN and TE | 2174 | Adherence, efficacy, HRU | Maybe (adherence) | |
| Sweet DE [53] | EM | EFV/TDF/FTC  RPV/TDF/FTC  EVG/COBI/TDF/FTC | g EFV+ TDF+ g3TC  RPV+TDF+g3TC  EVG/COBI+TDF+g3TC | TN | 200,000 | Cost, QALYs, ICER | No | |
| Daar et al [54] | RCT | EVG/c/TAF/FTC | EVG/c/TDF/FTC | TN | 203 | Efficacy,  safety/tolerability | No | |
| Wohl et al [55] | RCT | EVG/c/TAF/FTC | EVG/c/TDF/FTC | TN | 1733 | Safety/tolerability | No | |
| Wohl et al [56] | RCT | EVG/c/TAF/FTC | EVG/c/TDF/FTC | TN | 1733 | Efficacy,  safety/tolerability | No | |
| Gallant et al [40] | RCT | FTC/TAF + third agent | FTC + TDF+ third agent | TE | 663 | Efficacy,  safety/tolerability | No | |
| Biagi et al [57] | OS | EVG/COBI/TDF/FTC+ATV ± ABC | None | TE | 7 | Efficacy,  safety/tolerability | No | |
| Huhn et al [58] | RCT | EVG/c/TAF/FTC + DRV | Baseline MTR | TE | 135 | Adherence, efficacy, safety/tolerability, PRO | No | |
| Chen et al [59] | OS | STR | MTR single dose, MTR multi-dose | TE | 755 | Adherence, efficacy, PRO | Yes (adherence) | |
| Tennant et al [60] | OS | NNRTI-based STR | PI-based MTR | TE | 389 | Adherence, efficacy | Yes (adherence) | |
| Orkin 2017 [61] | RCT | DRV/c/TAF/FTC | Boosted PI + FTC/TDF | TE | 1141 | Adherence, efficacy, safety/tolerability, | Yes (adherence, efficacy, safety/tolerability) | |
| AMBER [62] | RCT | DRV/c/TAF/FTC | DRV/c + FTC/TDF | TN | 725 | Adherence, efficacy, safety/tolerability | No | |

Abbreviations: 3TC, lamivudine; ABC: abacavir; ABC/3TC: Kivexa®; ABC/AZT/3TC: Trizivir®; ART: Antiretroviral therapy; ATV/r: atazanavir/ritonavir; AZT: zidovudine; AZT/3TC: Combivir®; COBI: cobicistat; d4T: stavudine; DLT: dolutegravir; DLT+ABC+3TC: Triumeq®; DRV/r: darunavir/ritonavir; EFV: efavirenz; EFV/TDF/FTC: Atripla®; EVG: elvitegravir; EVG/COBI/TDF/FTC: Stribild®; FPV: Fosamprenavir; FTC: emtricitabine; LPV/r: lopinavir/ritonavir; NA: not applicable; NNRTI: non-nucleoside reversetranscriptase inhibitor; NRTIs: nucleoside reverse-transcriptase inhibitor; NVP: nevirapine; PI: protease inhibitor; RAL: raltegravir; RPV: Rilpivirine; RVP/TDF/FTC: Complera®/Eviplera®; SQV: Saquinavir; TE: treatment experienced; TN: treatment naive; TDF: tenofovir; TDF/FTC: Truvada®.

Note: x/y notation indicates single co-formulated drug containing both x & y; x+y notation indicates 2 separate drugs taken together. "2 NTRIs" usually included FDCs such as TDF/FTC, AZT/3TC or ABC/3TC.
