# Supplementary material for: A meta-analysis comparing 48-week treatment outcomes of single and multi-tablet antiretroviral regimens for the treatment of people living with HIV
Source: AIDS Res Ther. 2018 Oct 30;15:17. doi: 10.1186/s12981-018-0204-0 (PMC6206661; doi:10.1186/s12981-018-0204-0)
Supplement: Supplementary file 2 — Additional file 2. Characteristics of study population included in the meta-analysis. [file 12981_2018_204_MOESM2_ESM.docx]

**Table S2. Characteristics of study population included in the meta-analysis.**

| **Study** | **Study period** | **Country** | **Treatment regimen** | **N** | **Age, median (range)** | **Female n (%)** | **BL CD4 count/μl, n (%)  Mean (SD), Median (range)** | **BL Viral load (log) copies/ml** | **Median duration since first positive HIV-1 test (years), mean (SD)** | **Prior treatment, n(%)** | | **BL Comorbidity, n (%)** |
| --- | --- | --- | --- | --- | --- | --- | --- | --- | --- | --- | --- | --- |
|  |  |  |  |  |  |  |  |  |  | **Naïve** | **Experienced** |  |
| Arribas et al [1] | 2011 - 2012  96 weeks | Europe & North America | EVG/COBI/TDF/FTC | 293 | 41 yr (33–48) | 43 (15) | Mean: 604 (SD=275) | NR | 6 (4·8) | Not Appl. | Atazanavir: 123 (42); Darunavir: 113 (39); Lopinavir: 49 (17); Fosamprenavir: 6 (2); Saquinavir: 2 (1); | Positive HBsAg: 10 (3); Positive hepatitis C virus antibody: 19 (7) |
|  |  |  | TDF/FTC + RTV-boosted PI (ATV or DRV or LPV or FPV or SQV) | 145 | 40 yr (35–47) | 19 (14) | Mean: 624 (SD=270) | NR | 5 (3·6) | Not Appl. | Atazanavir: 51 (37); Darunavir: 60 (43); Lopinavir: 23 (16); Fosamprenavir:5 (4); Saquinavir:0 | Positive HBsAg: 3 (2); Positive hepatitis C virus antibody: 10 (7) |
| Bangsberg et al [15] | 1996 - 2008 6 months | USA | EFV/TDF/FTC | 47 | Mean (SD): 47.2 (8.2) | 10 (21.3) | NR | NR | NR | NR | ART: 25 (53.2) | NR |
|  |  |  | RTV-boosted PI + 2 NRTIs | 57 | Mean (SD): 44.3 (7.3) | 19 (33.3) | NR | NR | NR | NR | ART: 11 (19.3) | NR |
|  |  |  | NNRTI + 2 NRTIs | 14 | Mean (SD): 43.6 (8.6) | 21.4 (22) | NR | NR | NR | NR | ART: 6 (42.9) | NR |
| Buscher et al [17] | 18 months | USA | EFV/TDF/FTC | 34 | <30 yr old: 22 (22) 30–39 yr old: 35 (35) 40–49 yr old: 24 (24) 50 and above: 18 (18) | 27 (27) | 135 K/mm3 (36, 271) | 5.32 (4.90, 5.73) | NR | 29 | Not Applicable | NR |
|  |  |  | >1 pill, once daily regimen | 36 |  |  |  |  |  | 34 |  |  |
|  |  |  | >1 pill, twice-daily regimen | 29 |  |  |  |  |  | 36 |  |  |
| Dejesus et al [4] | 2006 48 weeks | USA & Puerto Rico | EFV/TDF/FTC | 203 | 43 (37–47) | 114 (56) | 517 (367-670) | <50: 96 % 50 to <200: 3 % ≥200: 1 % | NR | Not Appl. | PI: 108 (53); NNRTI: 95 (47) | NR |
|  |  |  | PI (with or without RTV boosting) + at least 2 NRTIs or NNRTI + at least 2 NRTIs | 97 | 43 (38–50) | 11 (11) | 515 (377-649) | <50: 98 % 50 to <200: 2 % ≥200: 0 % | NR | Not Appl. | PI: 52 (54); NNRTI: 45 (46) | NR |
| Fabbiani et al [21] | 1999 - 2012 | Italy | EFV+(TDF+FTC/3TC) | 96 | 42 (32-47) | 29 (30.20) | median (IQR): 430 (322-573) | Median (IQR):  4.8 (4.1-5.3) | Median (IQR): 3.0 (1.4-7.6) | 35 (36.5) | 61 (63.5) |  |
|  |  |  | EFV+(TDF+FTC/3TC) or EFV+(ABC+3TC) or EFV+(AZT+3TC) or EFV+(other) | 457 | 38 (33-45) | 133 (29.10) | median (IQR): 275 (185-433) | Median (IQR):  4.8 (4.6-5.3) | Median (IQR): 2.5 (0.5-7.4) | 248 (54.3) | 209 (45.7) |  |
| Palella et al [8] | 2010 - 2012 48 weeks | Europe & North America | RVP/TDF/FTC | 317 | 42 (35 - 48) | 44 (13.9) | Median: 576 (SD: 236.6) | NR | NR | NA | Atazanavir: 122 (38.5); Lopinavir: 97 (30.6); Darunavir: 63 (19.9); Fosamprenavir: 25 (7.9); Saquinavir: 6 (1.9); Amprenavir: 1 (0.3) | NR |
|  |  |  | RTV-boosted PI + two NRTIs | 159 | 43 (36 - 49) | 15 (9.4) | Median: 600 (SD: 258.8) | NR | NR | NA | Atazanavir: 54 (34.0); Lopinavir: 58 (36.5); Darunavir: 33 (20.8); Fosamprenavir: 12 (7.5); Saquinavir: 2 (1.3); Amprenavir: 0 (0) | NR |
| Pozniak et al [9] | 2011 - 2012 96 weeks | Australia, Europe & North America | EVG/COBI/TDF/FTC | 291 | 43 (34–49) | 23 (8) | Mean: 586 (SD:210) | NR | Mean: 6 (SD=4.3) | NA | Efavirenz: 232 (80); Coformulated efavirenz, emtricitabine, and tenofovir: 222 (76); Nevirapine: 47 (16); Rilpivirine: 9 (3); Coformulated rilpivirine, emtricitabine, and tenofovir: 7 (2); Etravirine: 3 (1) | Positive for surface antigen of the hepatitis B virus: 5 (2); Positive for hepatitis C virus antibody: 11 (4) |
|  |  |  | NNRTI (EFV & non-EFV) + TDF/FTC | 147 | 39 (32–48) | 9 (6) | Mean: 593 (SD:225) | NR | Mean: 5 (SD=2.9) | NA | EFV: 106 (74); EFV/TDF/FTC: 100 (70); Nevirapine: 27 (19); Rilpivirine: 10 (7); Coformulated rilpivirine, emtricitabine, and tenofovir: 9 (6); Etravirine: 0 | Positive for surface antigen of the hepatitis B virus: 3 (2); Positive for hepatitis C virus antibody: 2 (1) |
| Skwara et al [30] | 2013 6 months | Poland | EFV/TDF/FTC, RVP/TDF/FTC | 31 | 40 | 6 (19) | NR | NR |  | NR | NR | NR |
|  |  |  | Any cART (incl. FDCs), with >1 pills | 64 | 37.5 | 12 (19) | NR | NR |  | NR | NR | NR |
| Sterrantino et al [31] | 2010 - 2012 | Italy | EFV/TDF/FTC and PI-based and NNRTI-based regimen, with NRTI backbone (incl. FDCs) | 372 | 47.7 (42.1-54.5) | 85 (22.8) | Median (IQR): 602 (423.5-825) | <50 copies/ml: 334 (89.8) | NR | 53 (14.2) | Overall duration of ART (months), median (IQR): 123 (55.2-173); Duration of current cART (months), median (IQR): 19.2 (11.0-36.4) | Hepatitis C positive: 76 (20) |
| Choi et al [38] | 144 wk | USA, Europe, Thailand, Other | EVG/c/TDF/FTC | 23 | 33 (19-48) | 4 (17) | Mean: 374 (220-570) | Median (log10): 4.8 | NR | 100% | NA | NR |
|  |  |  | EFV/TDF/FTC | 10 | 32 (25-49) | 1 (10) | Mean: 338 (152-653) | Median (log10): 4.6 | NR |  |  |  |
|  |  |  | ATV + RTV + FTC/TDF | 17 | 35 (19-52) | 2 (12) | Mean: 346 (51-507) | Median (log10): 4.6 | NR |  |  |  |
|  | 96 wk |  | EVG/c/TDF/FTC | 11 | 33 (22-45),  45 (40-51) | 2 (29),  0 (0) | Mean: 548 (327-996),  Mean: 402 (210-805) | NR | NR | NA | NR | NR |
|  |  |  | PI + RTV + TVD | 2 | 48 (40-55) | 0 (0) | Mean: 478 (385-570) | NR | NR |  |  |  |
|  |  |  | NNRTI + TVD | 9 | 38 (26-50) | 0 (0) | Mean: 592 (300-927) | NR | NR |  |  |  |
| Rijinders et al [44] | 48 wk | NR | EVG/c/TAF/FTC | 402 | 41 | 56 (14) | Median: 659 | NR | NR | NA | ATV/r + FTC/TDF: 385 (64) ATV/c + FTC/TDF: 216 (36) | NR |
|  |  |  | ATV bosted + FTC/TDF | 199 | 41 | 29 (14) | Median: 633 | NR | NR | NA |  | NR |
| Chen et al [36] | 6 wk | US | STR | 166 | 21-72 | 217 (29.01) | NR | NR | 10-20 yr | NA | 100% | NR |
|  |  |  | MTR single-dose | 300 |  |  |  |  |  |  |  |  |
|  |  |  | MTR multi-dose | 284 |  |  |  |  |  |  |  |  |
| Sutton et al [52] | 2006 –2013 | US | STR | 580 | 43 (10) | 321 (55.3) | >350 cells/mm^3^: 197 (34.0) | ≤400 copies/ml: 164 (28.3) | NR | 485 (83.6) | 95 (16.4) | NR |
|  |  |  | MTR | 1594 | 42 (10) | 259 (44.7) | >350 cells/mm^3^: 625 (39.2) | ≤400 copies/ml: 461 (28.9) |  | 596 (37.4) | 998 (62.6) | NR |
| Orkin 2017 [61] | 48 wk | Multi-country | DRV/c/TAF/FTC | 763 | Mean (SD): 45.3 (10.86) | 140 (18.3) | >500 cells/mm3: 536 (70.2) | <50 copies/ml: 747 (97.9) | 11.76 (8.441) months | NA | 100% | NR |
|  |  |  | Boosted PI + FTC/TDF | 378 | Mean (SD): 44.8 (10.77) | 65 (17.2) | >500 cells/mm3: 260 (68.8) | <50 copies/ml: 371 (98.1) | 11.26 (8.181) months | NA | 100% | NR |
