# Supplementary material for: A meta-analysis comparing 48-week treatment outcomes of single and multi-tablet antiretroviral regimens for the treatment of people living with HIV
Source: AIDS Res Ther. 2018 Oct 30;15:17. doi: 10.1186/s12981-018-0204-0 (PMC6206661; doi:10.1186/s12981-018-0204-0)
Supplement: Supplementary file 3 — Additional file 3. a. Quality of RCTs Included in quantitative evidence synthesis (studies included in meta-analysis). b. Quality of observational studies included in quantitative evidence synthesis (studies included in meta-analysis). [file 12981_2018_204_MOESM3_ESM.docx]

**Table S3a.** **Quality of RCTs Included in quantitative evidence synthesis (studies included in meta-analysis)**

| **RCTs** | **Arribas et al. (2014)[1]** | **Dejesus et al. (2009)[4]** | **Palella et al.**  **(2014)[8]** | **Pozniak et al. (2014)[9]** | **Choi**  **(2016)[38]** | **Rijnders**  **(2015)[44]** | **Orkin**  **(2017)[61]** |
| --- | --- | --- | --- | --- | --- | --- | --- |
| **Selection bias** | | | | | | | |
| Was randomization carried out appropriately? | Yes | Unclear | Yes | Yes | Yes | Yes | Yes |
| Risk of bias | Low | Unclear | Low | Low | Low | Low | Low |
| Was the concealment of treatment allocation adequate? | Yes | Unclear | Yes | Yes | Yes | Yes | Yes |
| Risk of bias | Low | Unclear | Low | Low | Low | Low | Low |
| **Performance bias** | | | | | | | |
| Were the groups similar at the outset of the study in terms of prognostic factors, for example, severity of disease? | Yes | Yes | Yes | Yes | Yes | Yes | Yes |
| Risk of bias | Low | Low | Low | Low | Low | Low | Low |
| Were the care providers, participants and outcome assessors blind to treatment allocation? | No | No | No | No | No | No | No |
| Risk of bias | High | High | High | High | High | High | High |
| **Reporting bias** | | | | | | | |
| Is there any evidence to suggest that the authors measured more outcomes than they reported? | No | No | No | No | No | No | No |
| Risk of bias | Unclear | Low | Low | Low | Low | Low | Low |
| **Detection bias** | | | | | | | |
| Did the analysis include an intention-to-treat analysis? | Yes | Yes | Yes | Yes | Yes | Unclear | Yes |
| Appropriate? | Yes | Yes | Yes | Yes | Yes | Yes | Yes |
| Missing data handling | Yes | No | Yes | Yes | Yes | Yes | Yes |
| Risk of bias | Low | Unclear | Low | Low | Low | Low | Low |
| **Attrition bias** | | | | | | | |
| Were there any unexpected imbalances in drop-outs between groups? | No | No | No | No | No | Unclear | No |
| Were they explained or adjusted for? | Yes | Yes | Yes | Yes | Yes | Unclear | Yes |
| Risk of bias | Low | Low | Low | Low | Low | Unclear | Low |

**Table S3b. Quality of observational studies included in quantitative evidence synthesis (studies included in meta-analysis)**

| **CASP Section**  **Question** | **Fabbiani et al. (2014)[21]** | **Skwara et al. (2014)[30]** | **Buscher et al.  (2012)[17]** | **Bangsberg et al. (2010)[15]** | **Sterrantino et al. (2012)[31]** | **Chen**  **(2017)[59]** | **Sutton**  **(2016)[52]** |
| --- | --- | --- | --- | --- | --- | --- | --- |
| **Are the results of the study valid?** | | | | | | | |
| Did the study address a clearly focused issue? | Yes | Yes | Yes | Yes | Yes | Yes | Yes |
| Was the cohort recruited in an acceptable way? | Yes | Can’t tell | Yes | Yes | No | No | No |
| Was the exposure accurately measured to minimize bias? | Yes | Yes | Yes | Can't tell | Yes | No | No |
| Was the outcome accurately measured to minimize bias? | Yes | No | Yes | Yes | No | No | No |
| Have the authors identified all important confounding factors? | Yes | No | Yes | Can't tell | Yes | Yes | Yes |
| Have they taken account of the confounding factors in the design and/or analysis? | Yes | Can't tell | Yes | Yes | No | No | No |
| Was the follow up of subjects complete enough? | Yes | Yes | Yes. | Can't tell | Can't tell | No | No |
| Was the follow up of subjects long enough? | Yes | Yes | Yes | Yes | Can't tell | No | No |
| **What are the results?** | | | | | | | |
| What are the results of this study? | STR found to be associated with higher adherence, lower virological failure and low CNS toxicity compared to MTR. | STR was associated with improved adherence, quality of life and efficacy compared to MTR. | STR showed better adherence compared to MTR (twice daily) in both overall and ART naive population. However, the difference was not significant for STR vs. MTR (>1 pill regimen) for all population. | One-pill per day STR was associated with good adherence and viral suppression in a challenging population. | Non adherence was lower in the STR as compared to multi tablet regimen. | Participants taking STR were  more likely to be adherent than those taking a single-dose MTR and those taking a multi-dose MTR, with no difference  between the latter two. | STR found to be associated with higher adherence rates and a lower risk of hospitalization  in patients with HIV infection  and AIDS covered by South Carolina Medicaid  . |
| How precise are the results? | Results appear precise as confidence intervals were not so wide. | Results were not presented with the variance. | The study findings were precise as inter quartile ranges were found to be narrow. | Precision was unclear as study did not report results with the confidence intervals. | The study results were precise enough. | Results appear precise as confidence intervals were not so wide. | Results appear precise as confidence intervals were not so wide. |
| Do you believe the results? | Can't tell; Uncontrolled bias can occur in the retrospective studies. | Can't tell | Yes | Yes | Yes | Can't tell; Uncontrolled bias can occur in cohort studies. | Yes |
| **Will the results help locally?** | | | | | | | |
| Can the results be applied to the local population? | Yes | Yes | Yes | Yes | No | Yes | Yes |
| Do the results of this study fit with other available evidence? | Yes | Yes | No | Yes | Yes | Yes | Yes |
| What are the implications of this study for practice? | There was difference in baseline characteristics between the treatment groups so the results should be considered cautiously. | The results should be considered cautiously as the treatment duration was longer in patients using multiple-tablet regimens. | The study results could not be generalized as patients, who did not receive HAART during the study, died or was not followed up. | Simplification of therapy represents an important step forward in supporting adherence and treatment success. | The study overestimates the adherence, as patients were on steady cART; also self reporting may overestimate the level of adherence | The findings from this study have  implications for the design of adherence interventions. | Demonstrated the positive impact of STR on adherence and clinical outcome compared to MTR |
| Overall quality | Medium | Satisfactory | Medium | Satisfactory | Satisfactory | Medium | Satisfactory |
